# Supplementary material for: Relationships of temperature and biodiversity with stability of natural aquatic food webs
Source: Nat Commun. 2023 Jun 14;14:3507. doi: 10.1038/s41467-023-38977-6 (PMC10267189; doi:10.1038/s41467-023-38977-6)
Supplement: Supplementary file 1 — Supplementary Information File [file 41467_2023_38977_MOESM1_ESM.pdf]

Supplementary information

Supplementary figures

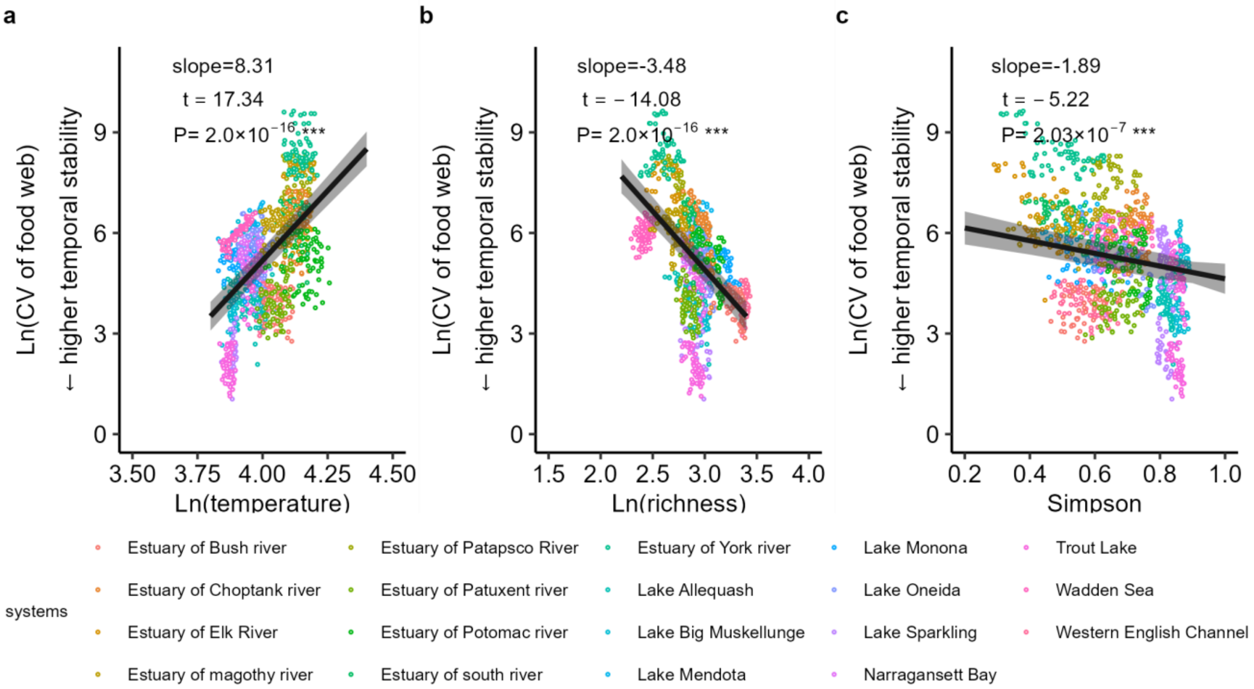

Figure S1. The effect of temperature, species richness and Simpson diversity on temporal stability (CV) of food web, by changing the width of time window to 3 years (12-time points). A smaller CV indicates a higher temporal stability. Coloured points (n=1644 biologically independent samples) correspond to values at each food web in each moving window (window width = 3 years). The bold black lines and error bands depict the significant best-fit trendline and the 95% confidence interval in the linear mixed model (two sided) across 19 food webs, respectively. The presented statistics are slopes, t values, and P values from the linear mixed models (two sided). These statistical values (slopes, t values, and P values) are the original ones from the best-fit linear mixed models (two sided), and no adjustments are made for multiple comparisons. Significant effects are indicated by *P <0.05, **P < 0.01, ***P < 0.001.

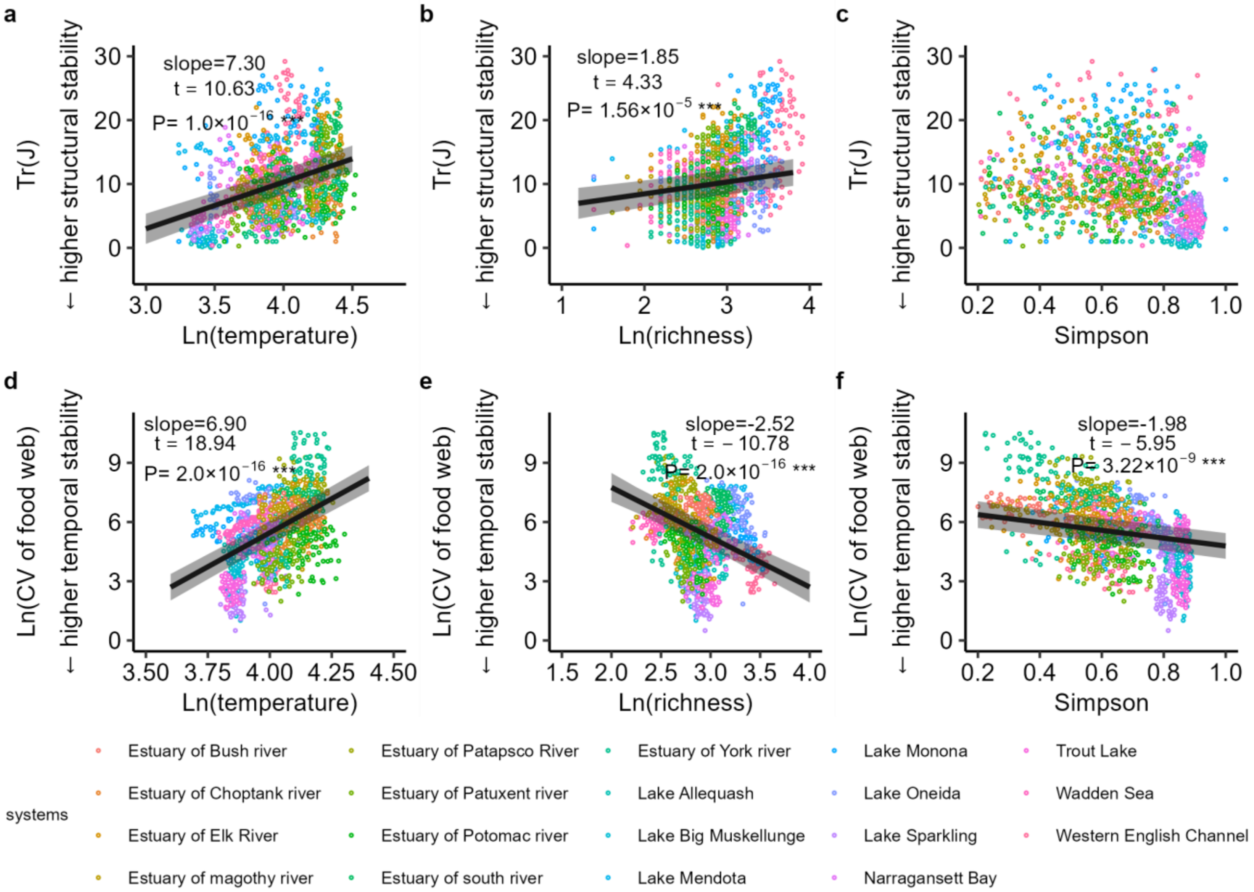

Figure S2. Inclusions of rare species into the analysis. As Fig. 2 in the main text, but including rare species into the analysis. In a−c, coloured points correspond to values at each food web in each season of each year (total points, n=1572 biologically independent samples). In d−f, coloured points (n= 1477) correspond to values at each food web in each moving window (window width = 1.5 years). In a-f, the bold black lines and error bands depict the significant best-fit trendline and the 95% confidence interval in linear mixed model (two-sided) across 19 food webs, respectively. The presented statistics are slopes, t values, and P values from the linear mixed models (two sided). These statistical values (slopes, t values, and P values) are the original ones from the best-fit linear mixed models (two sided), and no adjustments are made for multiple comparisons. Significant effects are indicated by *P <0.05, **P < 0.01, ***P < 0.001.

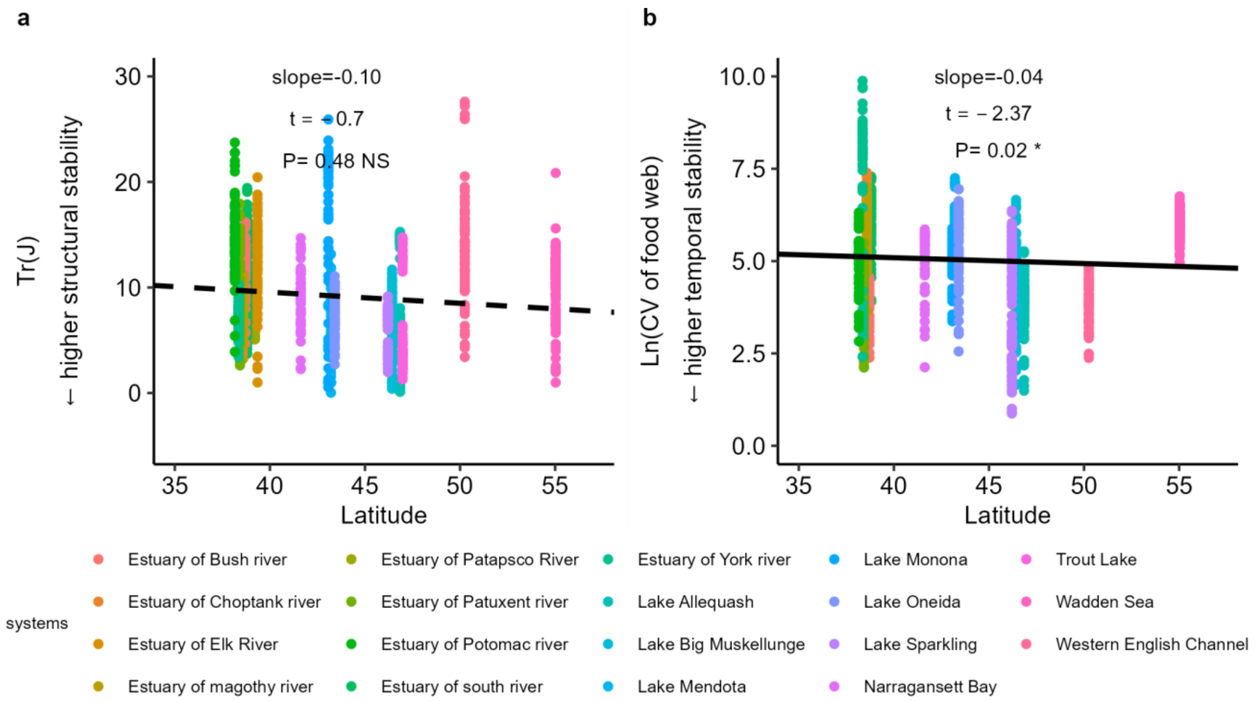

Figure S3. The effects of latitude on structural (a) and temporal stability of food web (b). In a, a linear mixed model (two sided) is employed to test for the effect of latitude on structural stability (Tr) of food web, by treating the year, season, and sampling locations (e.g. lake Mendota and Trout Lake) as random factor. Coloured points correspond to values at each food web in each season of each year (total points, n=1572 biologically independent samples). In b, a linear mixed model (two sided) is again employed to test effect of latitude on temporal stability (CV) of food web, by only treating the sampling locations (e.g. lake Mendota and Trout Lake) as random factor. Because temporal stability is calculated using a moving window, the year and season were factored out. Coloured points (n= 1477) correspond to values at each food web in each moving window (window width = 1.5 years). In a-b, the solid black lines depict the significant best-fit trendline in the linear mixed model (two sided) across 19 food webs. The presented statistics are slopes, t values, and P values from the linear mixed models (two sided). These statistical values (slopes, t values, and P values) are the original ones from the best-fit linear mixed models (two sided), and no adjustments are made for multiple comparisons. Significant effects are indicated by $^*P < 0.05$, $^{**}P < 0.01$, $^{***}P < 0.001$. NS indicates non-significant effects.

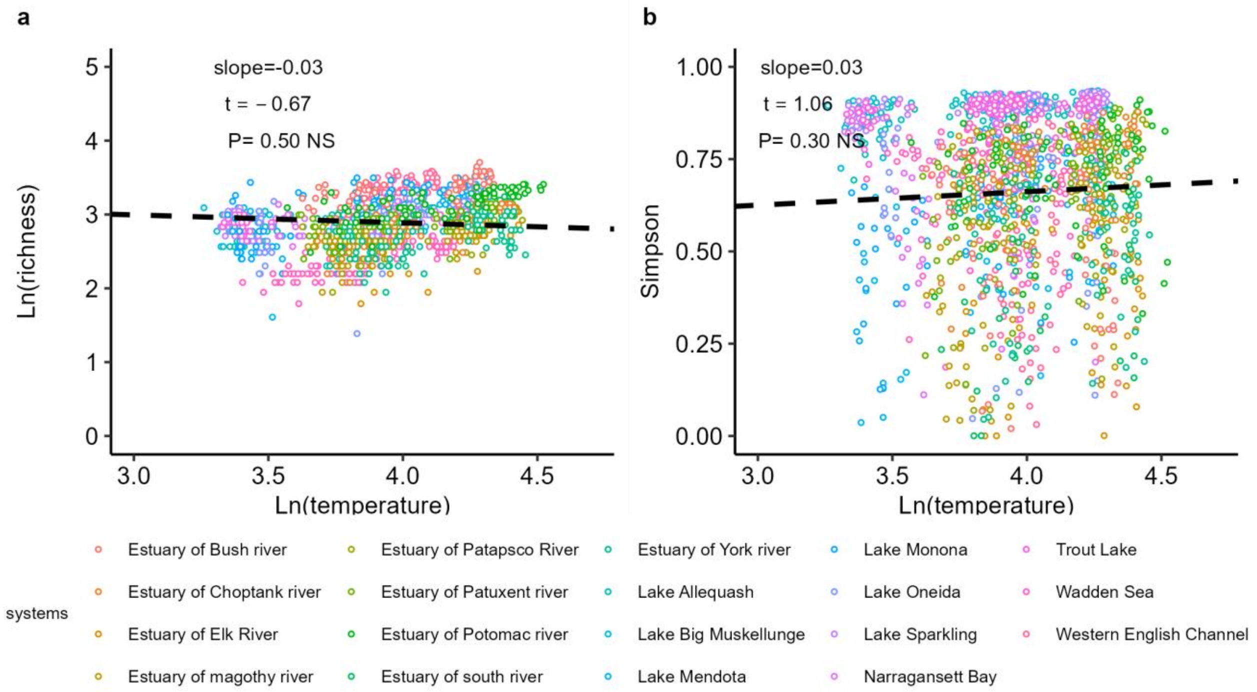

Figure S4. The effect of temperature on species richness (a) or Simpson diversity (b) across 19 studied food webs. Linear mixed models (two sided) are employed to test for the effects of temperature on species richness or Simpson, via the year, season, and sampling locations (e.g. lake Mendota and Trout Lake) as a random factor. Coloured points correspond to values at each food web in each season of each year (total points, n=1572 biologically independent samples). In a-b, the black dashed lines depict the best-fit trendline in the linear mixed model (two sided) across 19 food webs. The presented statistics are slopes, t values, and P values from the linear mixed models (two sided). These statistical values (slopes, t values, and P values) are the original ones from the best-fit linear mixed models (two sided), and no adjustments are made for multiple comparisons. Significant effects are indicated by *P <0.05, **P < 0.01, ***P < 0.001. NS indicates non-significant effects.

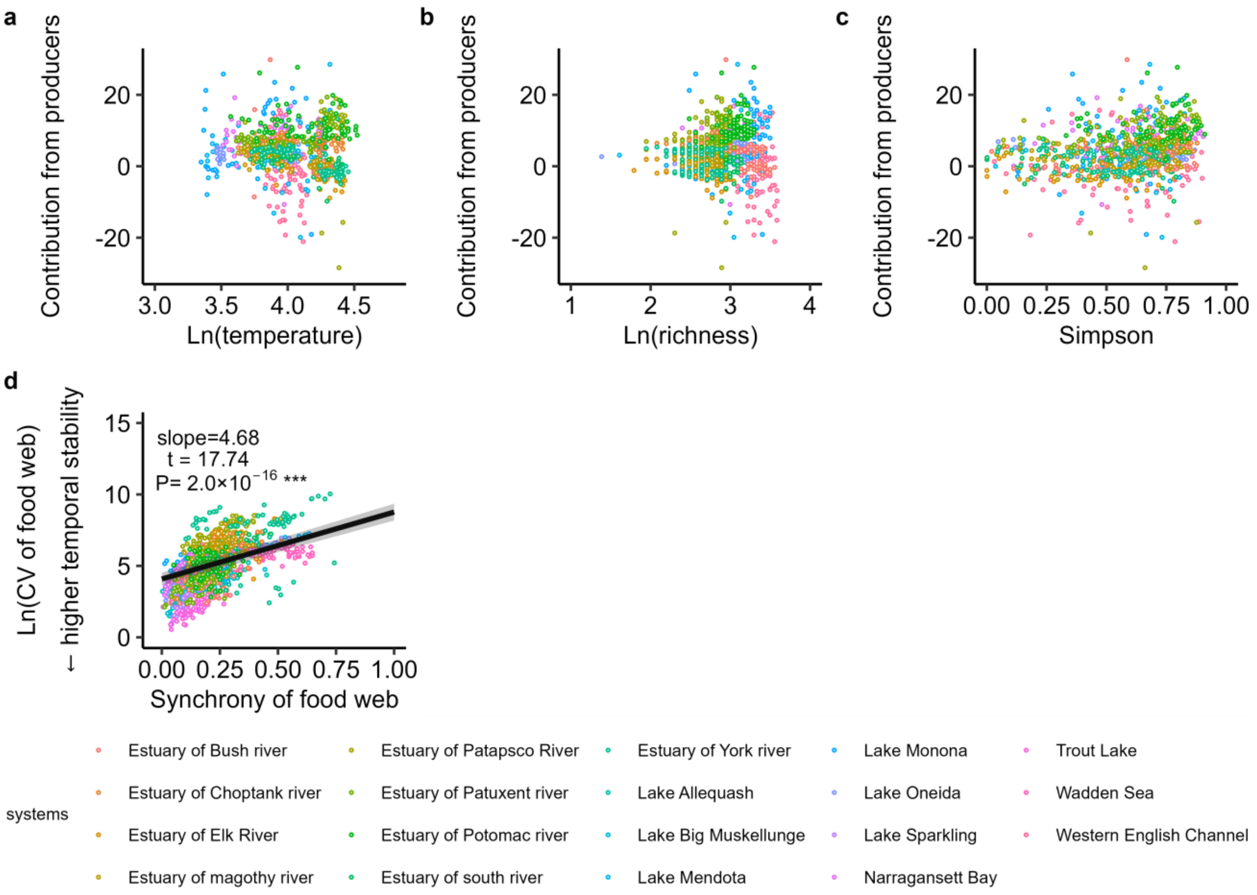

Figure S5. The effect of temperature, species richness and Simpson diversity on the contribution from producers to structural stability (a-c), and the effect of the synchrony of all species within the food web on temporal stability (d; coefficient of variation of species abundance CV) of food webs. In a−c, coloured points correspond to values of each food web in each season of each year (total points, n=1572 biologically independent samples). In d, coloured points (n= 1477) correspond to values at each food web in each moving window (window width = 1.5 years). In a-d, the bold black lines and error bands depict the significant best-fit trendline and the 95% confidence interval in the linear mixed model (two sided) across 19 food webs, respectively. The presented statistics are slopes, t values, and P values from the linear mixed models (two sided). These statistical values (slopes, t values, and P values) are the original ones from the best-fit linear mixed models (two sided), and no adjustments are made for multiple comparisons. Significant effects are indicated by *P <0.05, **P < 0.01, ***P < 0.001.

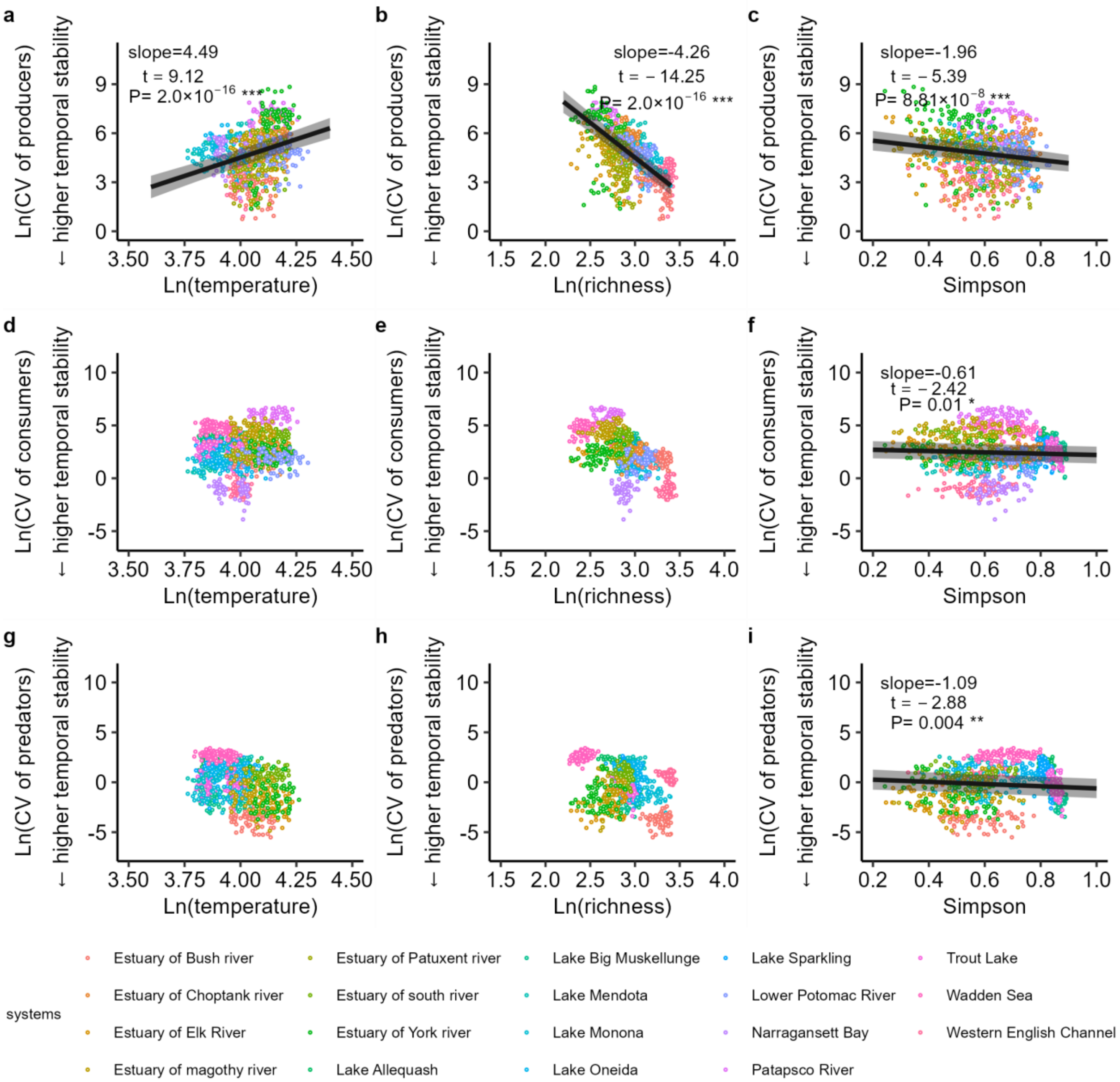

Figure S6. The effect of temperature, species richness, and Simpson diversity on the temporal stability (coefficient of variation of species abundance CV) of producers (a-c), consumers (d-f), and predators (g-i). In a−i, coloured points (n= 1477 biologically independent samples) correspond to values at each food web in each moving window (window width = 1.5 years). In a-i, the bold black lines and error bands depict the significant best-fit trendline and the 95% confidence interval in the linear mixed model (two sided) across 19 food webs, respectively. The presented statistics are slopes, t values, and P values from the linear mixed models (two sided). These statistical values (slopes, t values, and P values) are the original

ones from the best-fit linear mixed models (two sided), and no adjustments are made for multiple comparisons. Significant effects are indicated by *P <0.05, **P < 0.01, ***P < 0.001.

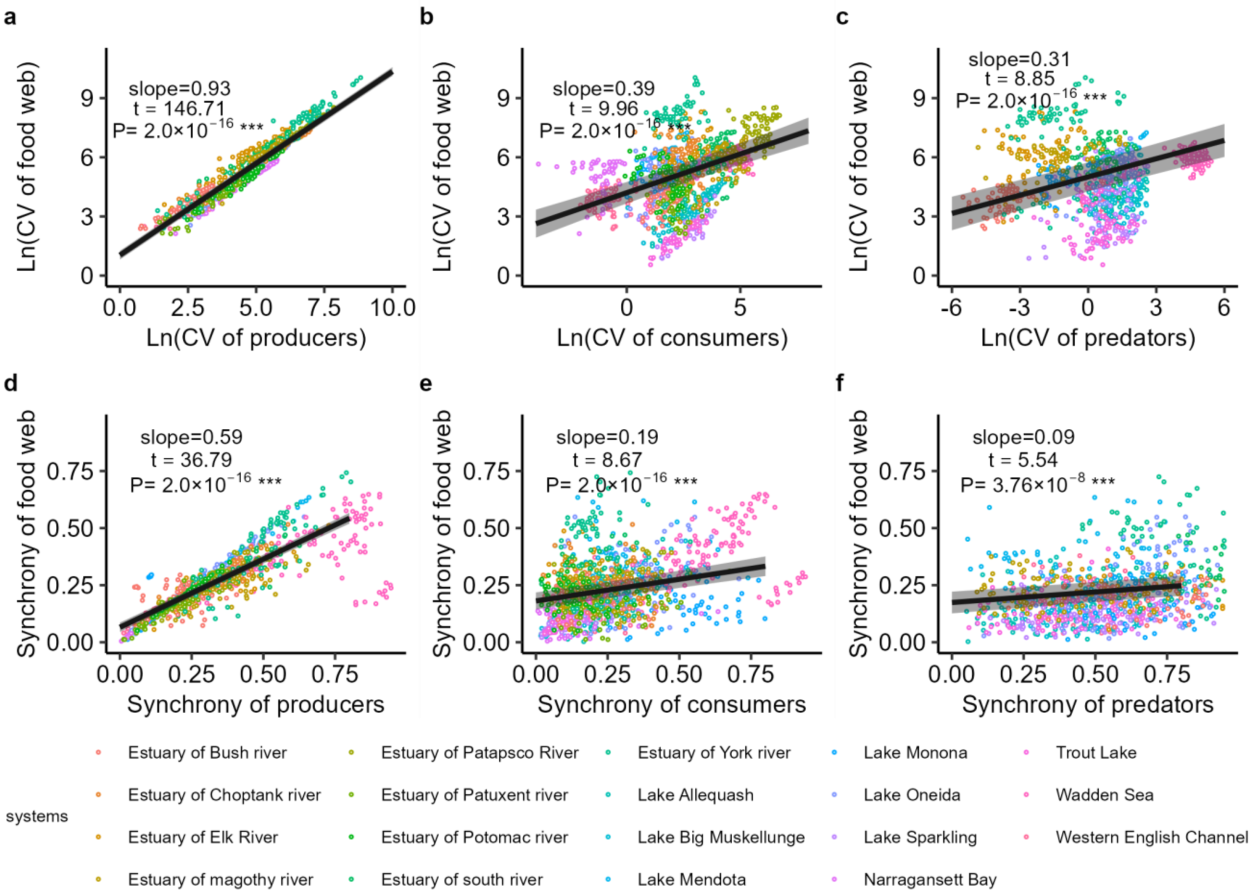

Figure S7. Contribution of each trophic group (producers, consumers, or predators) on temporal stability (CV) of whole food web (a-c), and contribution of each trophic group (producers, consumers, or predators) on synchrony of all species within the food web (d-f). In a-c, a linear mixed model (two sided) is employed to test for the effect of CV of each trophic group (producers, consumers, or predators) on CV of whole food web, by treating sampling locations (e.g. lake Mendota and Trout Lake) as random factor. In d-f, similarly, linear mixed model (two sided) is employed to test the effect of synchrony of each trophic group (producers, consumers, or predators) on synchrony of whole food web, by treating sampling locations (e.g. lake Mendota and Trout Lake) as random factor. Because temporal stability CV and synchrony is calculated using a moving window, the year and season were factored out. In a-f, coloured points (n= 1477 biologically independent samples) correspond to values in each

moving window (window width = 1.5 years, which is same length in main text Fig 2). The bold black lines and error bands depict the significant best-fit trendline and the 95% confidence interval in the linear mixed model (two sided) across 19 food webs, respectively. The presented statistics are slopes, t values, and P values from the linear mixed models (two sided). These statistical values (slopes, t values, and P values) are the original ones from the best-fit linear mixed models (two sided), and no adjustments are made for multiple comparisons. Significant effects are indicated by *P <0.05, **P < 0.01, ***P < 0.001.

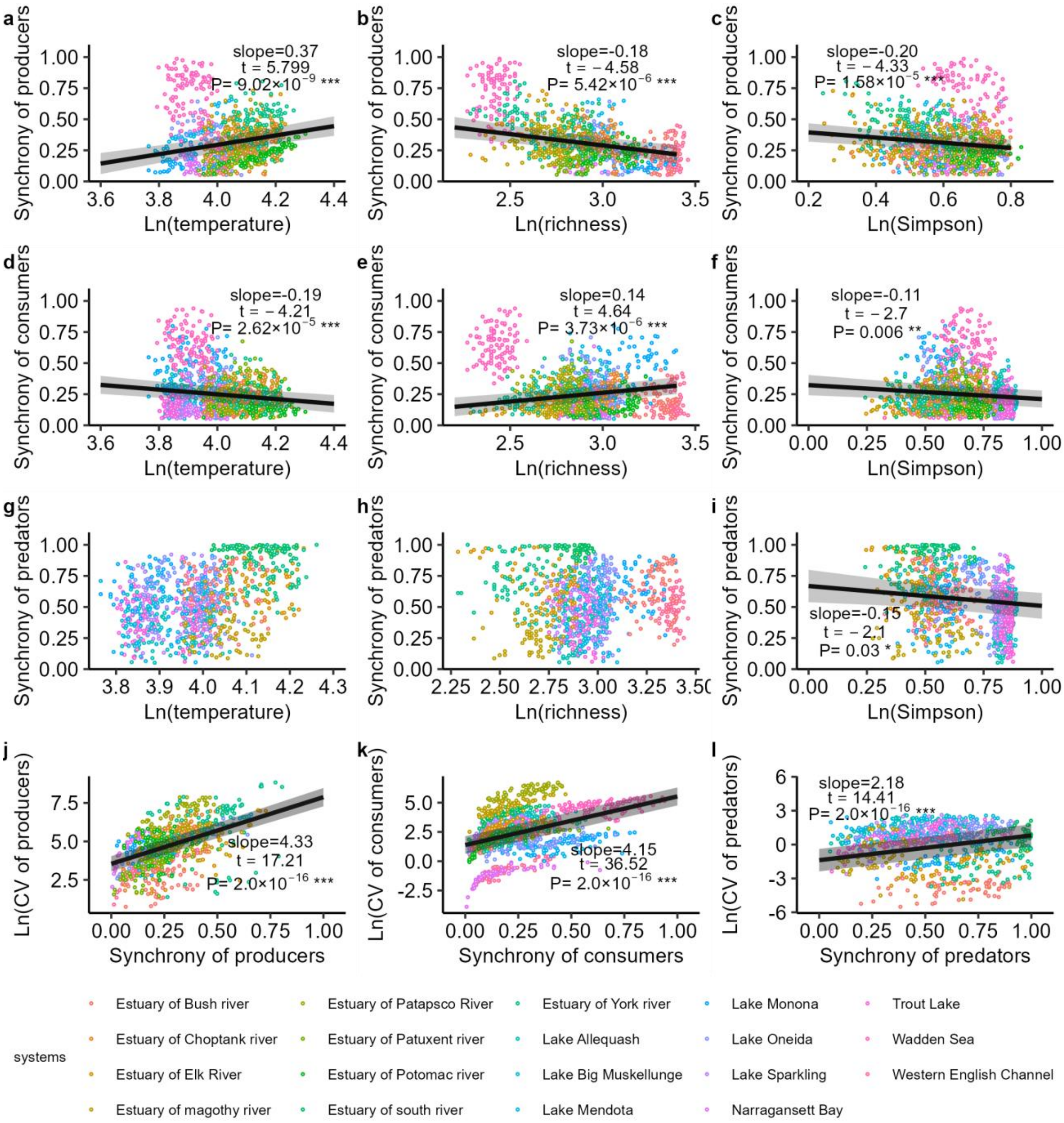

Figure S8. The effect of temperature, species richness, and Simpson diversity on synchrony of producers (a-c), consumers (d-f), and predators (g-i), and contribution of each trophic group (producers, consumers, or predators) on temporal stability (CV) of each trophic group (j-l). In a-c, a linear mixed model (two sided) is employed to test for effects of temperature, species richness, and Simpson diversity on synchrony of producers, consumers, and predators, by treating sampling locations (e.g. lake Mendota and Trout Lake) as a random factor. In d-f, a linear mixed model (two sided) is employed to test for the effect of

synchrony of each trophic group (producers, consumers, or predators) on CV of each trophic group, by treating sampling locations (e.g. lake Mendota and Trout Lake) as a random factor. Because temporal stability (CV) and synchrony is calculated using a moving window, the year and season are factored out. In a-l, coloured points (n= 1477 biologically independent samples) correspond to values in each moving window (window width = 1.5 years, which is same length in main text Fig 1). The bold black lines and error bands depict the significant best-fit trendline and the 95% confidence interval in the linear mixed model (two sided) across 19 food webs, respectively. The presented statistics are slopes, t values, and P values from the linear mixed models (two sided). These statistical values (slopes, t values, and P values) are the original ones from the best-fit linear mixed models (two sided), and no adjustments are made for multiple comparisons. Significant effects are indicated by *P <0.05, **P < 0.01, ***P < 0.001.

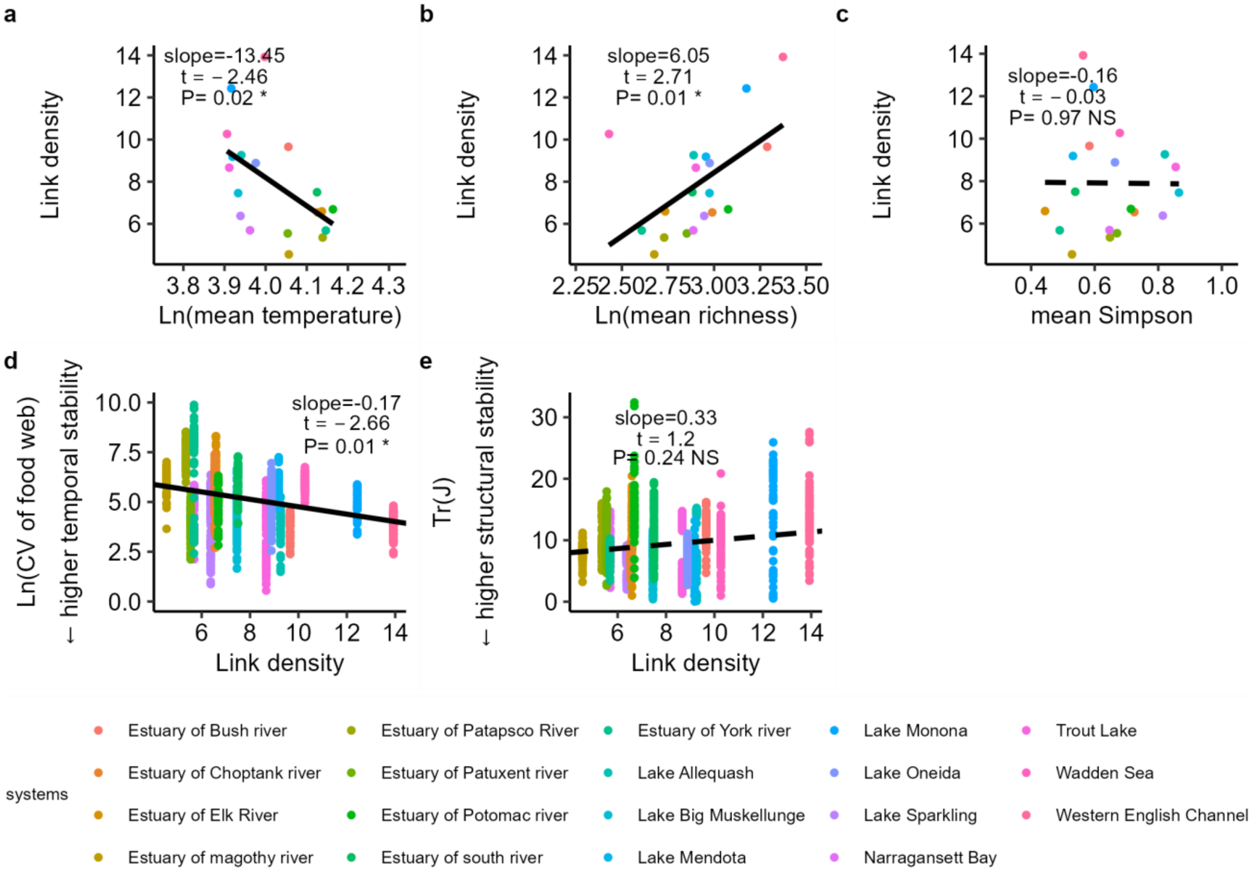

Figure S9. The effect of temperature, species richness, Simpson diversity on link density L/S across food webs (a-c), and the effects of link density on temporal and structural

stability of food web (d-e). In a-c, a linear regression (two sided) is employed to test for effects of geometric mean temperature, geometric mean richness and geometric mean Simpson on the links per species. Because link density in each food web is fixed value over time, we compute geometric mean temperature, geometric mean richness and geometric mean Simpson and treat them as explanatory variables in the linear regression model. Coloured points (total points, n= 19) represent biologically independent sample sites. The black solid lines depict the significant best-fit trendline in the linear model (two sided) across 19 food webs. In d-e, linear mixed models (two sided) are employed to test for the effects of link density on temporal or structural stability of food web, via treating sampling locations (e.g. lake Mendota and Trout Lake) as random factor. The solid black lines depict the significant best-fit trendline in the linear mixed model (two sided) across 19 food webs. In d, coloured points (total points, n=1477 biologically independent samples) correspond to values at each food web in each moving window (window width =1.5 years). In e, coloured points correspond to values at each food web in each season of each year (n=1572 biologically independent samples). In a-e, the presented statistics are slopes, t values, and P values from the linear model or linear mixed models (two sided). These statistical values (slopes, t values, and P values) are the original ones from the best-fit linear models or linear mixed models (two sided), and no adjustments are made for multiple comparisons. Significant effects are indicated by *P <0.05, **P < 0.01, ***P < 0.001. NS indicates non-significant effects.

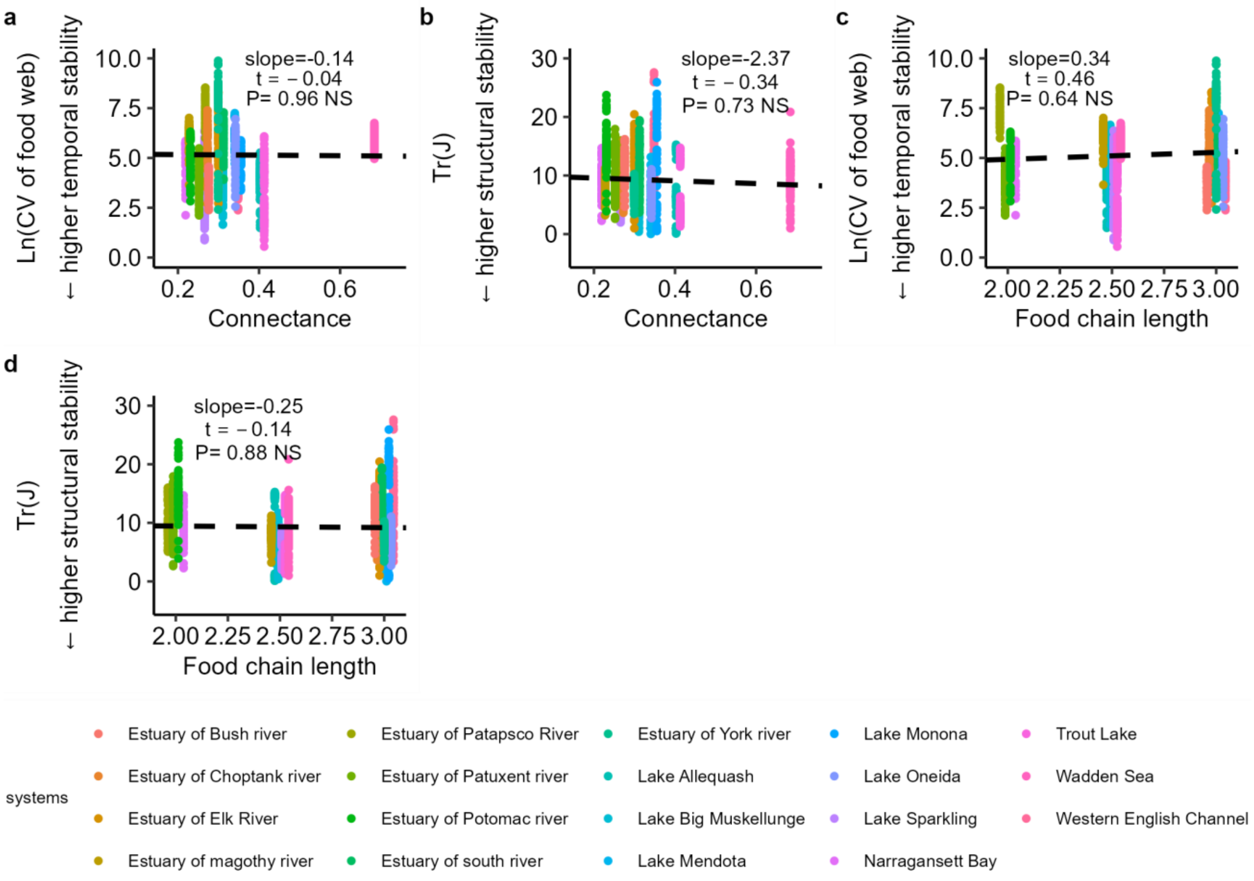

Figure S10. The effect of connectance L/S^2 and food chain length on temporal stability of food web (a, c), and structural stability (b, d) of food web. Linear mixed models (two sided) are employed to test for effects of connectance and food chain length on each of stability index (temporal or structural stability), via treating sampling locations (e.g. lake Mendota and Trout Lake) as random factor. The black dashed lines depict the best-fit trendline in the linear mixed model (two sided) across 19 food webs. In b and d, coloured points correspond to values at each food web in each season of each year (total points, n=1572 biologically independent samples). In a and c, coloured points (n= 1477) correspond to values at each food web in each moving window (window width = 1.5 years). In a-d, the presented statistics are slopes, t values, and P values from the linear mixed models (two sided). These statistical values (slopes, t values, and P values) are the original ones from the best-fit linear mixed models (two sided), and no adjustments are made for multiple comparisons. Significant effects are indicated by *P <0.05, **P < 0.01, ***P < 0.001. NS indicates non-significant effects.

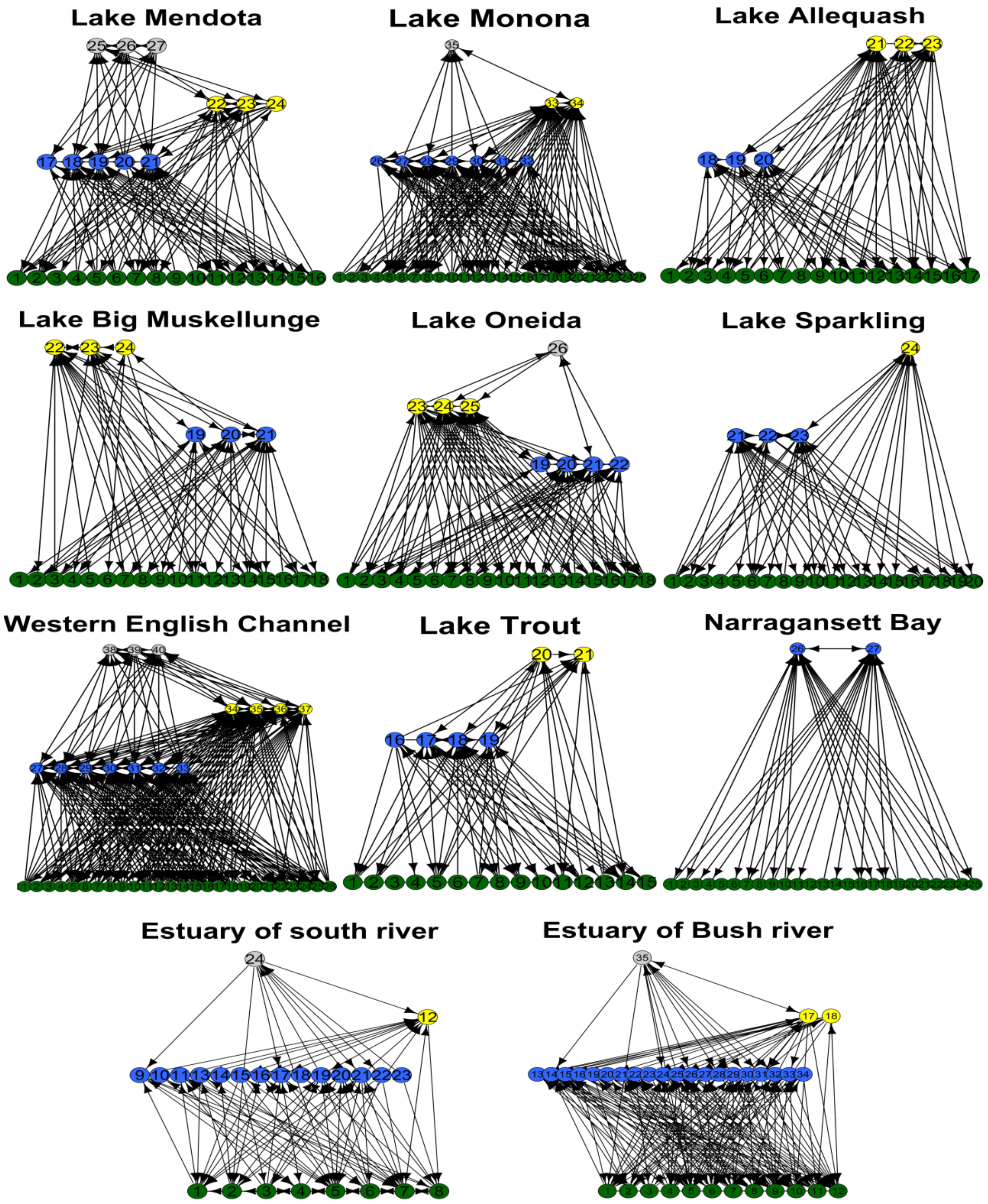

Figure S11. Interaction networks from 11 out of 19 natural food webs. Arrows indicate species interactions identified by CCM. The number within circles shows species identity (see References 1[1]). Circle colours indicate trophic levels. Interaction networks from 8 out of 19 natural food webs see Figure S12.

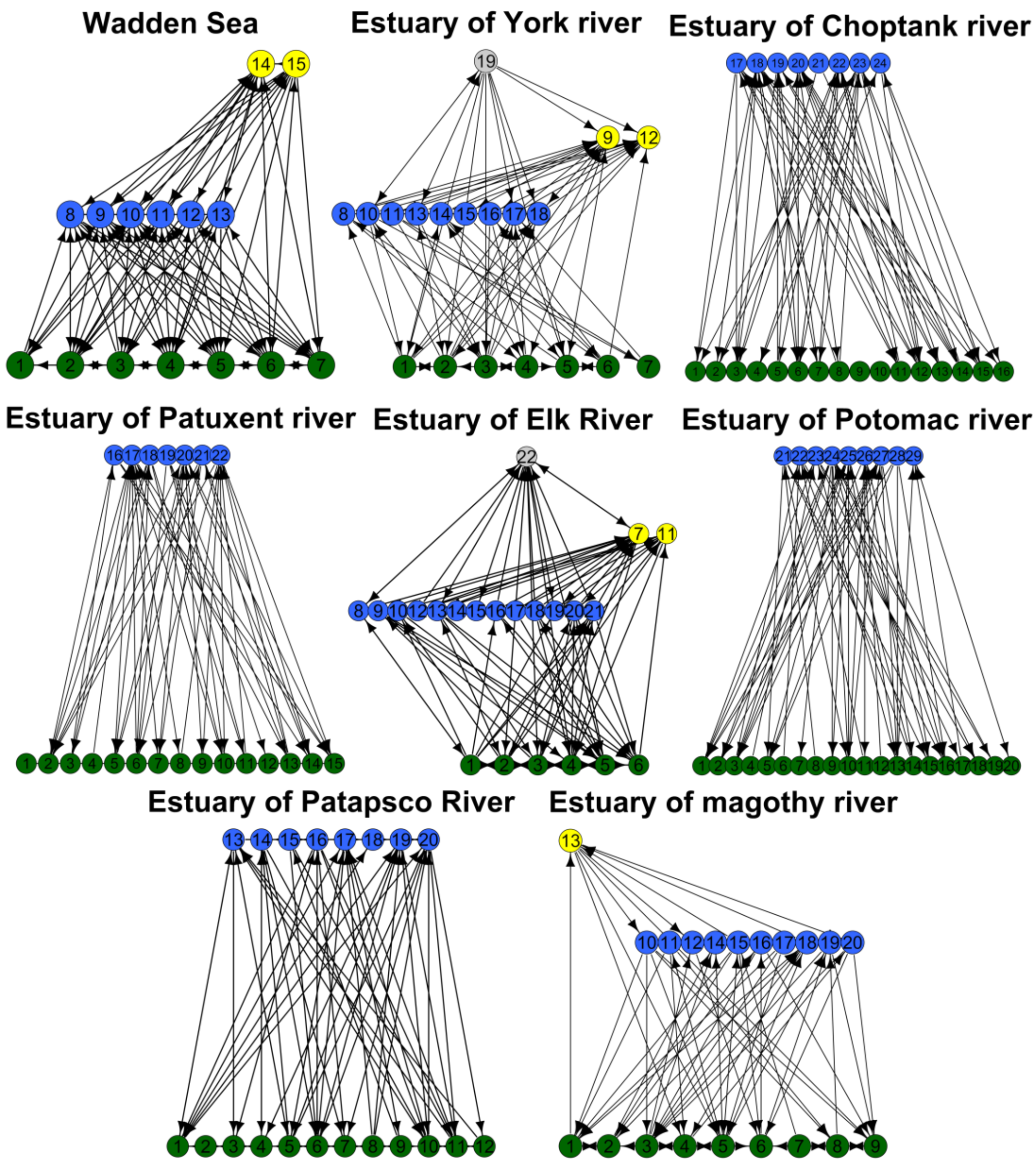

Figure S12. Interaction networks from 8 out of 19 natural food webs. Arrows indicate species interactions identified by CCM. Number within circles reveal species identity (see References 1[1]). Circle colours indicate trophic level.

Supplementary Information

The aim of this section is to schematically illustrate contribution of two trophic groups (predators and consumers) on structural stability $\mathrm{Tr(J)}$. Assuming a food web, for any species i in this food web, species abundance x_i follow an implicit equation,

$$x_i(t+1) = x_i(t)(r_i + g_i(x_1, \dots, x_n)) \quad (1)$$

Here $(r_i + g_i(x_1, \dots, x_n))$ is the per capita growth rate, which is summation of the r_i (intrinsic growth rate) and the g_i (the effects of species $1, \dots, n$ on species i).

The structural stability $\mathrm{Tr(J)}$ of the food web is equivalent to trace of the Jacobian matrix J, written as,

$$\mathrm{Tr(J)} = \sum_i^n (r_i + x_i \frac{\partial g_i}{\partial x_i} + g_i) \quad (2)$$

Part 1. Contribution from predators

For predator species, the intrinsic growth rate r_i is generally negative. The $x_i \frac{\partial g_i}{\partial x_i}$ represents self-limitation (e.g. cannibalism). The g_i is the effects of consumers j on the predator, which can be written as $\alpha_{ij} x_j$ in case g_i is linear in x_j. The $\alpha_{ij} x_j$ (i.e. g_i) can in turn be written as $e\alpha_{ji} x_j$, where e is the conversion coefficient and α_{ji} is the predators' consumption rate. If g_i is nonlinear (i.e. Holling type II and III function), g_i can be in turn written as $e\frac{a{x_j}^m}{1+ah{x_j}^m}$ $(m \geq 1)$, where the $\frac{a{x_j}^m}{1+ah{x_j}^m}$ again represents the predators' consumption rate.

The contribution from predators is that part of $\mathrm{Tr(J)}$ representing predators (i.e. the sum of those diagonal elements that belong to predators). We found that temperature mostly increased this contribution (Fig. 3a in main text). This can happen when temperature: 1) increases g_i by increasing the predators' consumption rate (α_{ji} or $\frac{a{x_j}^m}{1+ah{x_j}^m}$); or 2) decreases the predator's intrinsic growth rate r_i; or 3) decreases the predators' self-limitation $x_i \frac{\partial g_i}{\partial x_i}$; or 4) increases the predators' consumption rate 1) to a greater extent than the other parameters 2)-3).

Part 2. Contribution from consumers

For consumers, r_i is generally negative too, and $x_i \frac{\partial g_i}{\partial x_i}$ again is self-limitation. The g_i is the sum of the following two parts: 1) effects of producers j on the consumer $e\alpha_{ji} x_j$ (i.e. the α_{ji} is consumers' consumption rate when g_i is linear); and 2) the effects of predators k on the consumer $\alpha_{ik} x_k$ (α_{ik} is predators' consumption rate when g_i is linear). If g_i is nonlinear, these

two parts are $e\frac{a{x_j}^m}{1+ah{x_j}^m}$ and $e\frac{a{x_i}^m}{1+ah{x_i}^m}$, where the $\frac{a{x_j}^m}{1+ah{x_j}^m}$ and $\frac{a{x_i}^m}{1+ah{x_i}^m}$ again represent the consumers' consumption rate and the predators' consumption rate, respectively.

We found that species richness increased the contribution from consumers (Fig. 3e in main text). This can happen when species richness: 1) increases g_i by increasing the consumers' consumption rate (α_{ji} or $\frac{a{x_j}^m}{1+ah{x_j}^m}$); or 2) decrease the predators' consumption rate (α_{ik} or $\frac{a{x_i}^m}{1+ah{x_i}^m}$); or 3) decreases the consumer's intrinsic growth rate r_i; or 4) decreases the consumers' self-limitation $x_i\frac{\partial g_i}{\partial x_i}$; or 5) increases the consumers' consumption rate 1) to a greater extent than the other parameters 2)-4).

Supplementary Tables

Table S1. Linear mixed models (two-sided) were applied to 1) test the effect of temperature, species richness and Simpson diversity on structural stability $\mathrm{Tr(J)}$, and components of $\mathrm{Tr(J)}$ from two trophic groups (i.e. predators and consumers), via treating the year, season, and sampling locations (e.g. lake Mendota and Trout Lake) as a random effects to exclude the potential confounding effect of them; 2) test the effect of temperature, species richness, and Simpson diversity on log-transferred temporal stability (CV) of whole food web and synchrony of all species within the food web, via treating the sampling locations as a random effect. The statistical values (slopes, t values, and P values) were the original ones from the best-fit linear mixed models (two sided), and no adjustments were made for multiple comparisons. Significant effects are indicated by *P <0.05, **P < 0.01, ***P < 0.001. NS indicated non-significant effects.

	Ln(temperature)			Ln(richness)			Simpson		
	slope	t value	P value	slope	t value	P value	slope	t value	P value
**1.**Structural stability of food web ($\mathrm{Tr(J)}$)	6.55	8.66	2.36×10^{-14} ***	3.02	6.69	3.10×10^{-11} ***	0.22	0.43	0.66 NS
**2.**Components of structural stability ($\mathrm{Tr(J)}$) from predators	23.94	3.24	0.0012 **	-11.52	-1.59	0.11 NS	14.32	1.47	0.14 NS
**3.**Components of structural stability ($\mathrm{Tr(J)}$) from consumers	-0.21	-0.07	0.94 NS	12.32	3.71	0.0003 ***	-5.16	-1.18	0.23 NS
**4.**Ln (CV of whole food web)	7.47	20.28	2.00×10^{-16} ***	-3.88	-15.76	2.00×10^{-16} ***	-2.13	-6.30	4.04×10^{-10} ***
**5.**Synchrony of whole food web	0.28	7.51	1.11×10^{-13} ***	-0.14	-5.85	7.12×10^{-9} ***	-0.19	-5.68	1.62×10^{-8} ***

Table S2. Data sources for food webs. The seasonal length shows the average length of time series, in which species abundance is averaged to the seasonal frequency (trimonthly) from corresponding raw data, and then then be used in main text. The raw length shows the length of time series species abundance, prior to seasonal averaging.

	Name	Year	Continent	Latitude	Longitude	Seasonal length	Raw length	Source
1	Lake Sparkling	1983-2011	North America	46.0054	-89.7029	116	318	[2–5]
2	Trout Lake	1983-2011	North America	46.0788	-89.6655	116	318	[2–5]
3	Western English Channel	1995-2015	Europe	50.2501	-4.2167	83	504	[6,7]
4	Estuary of Patapsco River	1984-2002	North America	39.2130	-76.5225	74	220	[8–10]
5	Estuary of Elk River	1984-2002	North America	39.4427	-76.0006	73	221	[8–10]
6	Lake Allequash	1983-2011	North America	46.0451	-89.6295	116	318	[2–5]
7	Lake Oneida	1975-1995	North America	43.2072	-75.9227	82	198	[11–13]
8	Narragansett Bay	1972-1986	North America	41.6238	-71.3528	60	179	[14–16]
9	Lake Big Muskellunge	1983-2011	North America	46.0239	-89.6001	116	317	[2–5]
10	Lake Mendota	1995-2010	North America	43.1113	-89.4255	64	162	[17–20]
11	Lake Monona	1995-2010	North America	43.0744	-89.3545	64	161	[17–20]
12	Estuary of south river	1984-2002	North America	38.9043	-76.4805	73	218	[8–10]
13	Estuary of Choptank river	1984-2002	North America	38.5807	-76.0587	74	220	[8–10]
14	Estuary of Bush river	1984-2002	North America	39.3609	-76.2537	72	214	[8–10]
15	Estuary of magothy river	1984-2002	North America	39.0480	-76.4070	74	219	[8–10]
16	Estuary of Patuxent river	1984-2002	North America	38.3127	-76.4550	74	220	[8–10]
17	Estuary of York river	1984-2002	North America	37.2345	-76.4234	74	219	[8–10]
18	Estuary of Potomac river	1984-2002	North America	38.1576	-76.598	74	221	[8–10]
19	Wadden Sea	1984-2011	North America	55.0300	8.4600	112	1016	[21,22]

Table S3. Pearson's correlation between Simpson and Shannon diversity in 19 food webs.

	Food web	Correlation		Food web	Correlation
1	Western English Channel	0.96	11	Estuary of Elk River	0.97
2	Trout Lake	0.95	12	Estuary of South river	0.96
3	Lake Allequash	0.94	13	Estuary of Choptank river	0.94
4	Lake Big Muskellunge	0.95	14	Estuary of Bush river	0.97
5	Lake Sparkling	0.97	15	Estuary of Magothy river	0.98
6	Narragansett Bay	0.96	16	Estuary of Patuxent river	0.96
7	Lake Monona	0.91	17	Estuary of York river	0.98
8	Lake Oneida	0.97	18	Estuary of Potomac river	0.97
9	Lake Mendota	0.97	19	Wadden Sea	0.97
10	Estuary of Patapsco River	0.95			

References

1. Zhao, Q. *et al.* Relationships of temperature and biodiversity with stability of natural aquatic food webs. Zenodo. https://doi.org/10.5281/zenodo.7877806 (2023).

2. O'Keefe, T. C. & Dodson, S. I. Long-term population dynamics of zooplankton in northern temperate lakes. *Int. Vereinigung für Theor. und Angew. Limnol. Verhandlungen* **27**, 1943–1945 (2000).

3. Martin, B. E., Walsh, J. R. & Vander Zanden, M. J. Rise of a native apex predator and an invasive zooplankton cause successive ecological regime shifts in a North Temperate Lake. *Limnol. Oceanogr.* **67**, S163–S172 (2022).

4. Martin, B. E., Mrnak, J. T. & Vander Zanden, M. J. Evaluating the potential role of predation by native fish regulating the abundance of invasive spiny water flea. *J. Freshw. Ecol.* **38**, (2023).

5. Magnuson, J., Carpenter, S. & Stanley, E. North Temperate Lakes LTER: Zooplankton - Trout Lake Area 1982 - current ver 37. Environmental Data Initiative. https://doi.org/10.6073/pasta/aba16e9867e8f7b41b08dae0e92d6a98 (2022).

6. Widdicombe, C. E., Eloire, D., Harbour, D., Harris, R. P. & Somerfield, P. J. Long-term phytoplankton community dynamics in the Western English Channel. *J. Plankton Res.* **32**, 643–655 (2010).

7. Reygondeau, G., Molinero, J. C., Coombs, S., MacKenzie, B. R. & Bonnet, D. Progressive changes in the Western English Channel foster a reorganization in the plankton food web. *Prog. Oceanogr.* **137**, 524–532 (2015).

8. Harding, L. W. *et al.* Long-Term Trends of Nutrients and Phytoplankton in Chesapeake Bay. *Estuaries and Coasts* **39**, 664–681 (2016).

9. Slater, W. L. *et al.* Jellyfish : How Does Hypoxia Impact the Chesapeake Bay Zooplankton Community? *Diversity* **12**, 1–26 (2020).

10. Marshall, H. G., Burchardt, L. & Lacouture, R. A review of phytoplankton composition within Chesapeake Bay and its tidal estuaries. *J. Plankton Res.* **27**, 1083–1102 (2005).

11. Schaffner, L. R. *et al.* Consumer-resource dynamics is an eco-evolutionary process in a natural plankton community. *Nat. Ecol. Evol.* **3**, 1351–1358 (2019).

12. Karatayev, V. A., Karatayev, A. Y., Burlakova, L. E. & Rudstam, L. G. Eutrophication and Dreissena invasion as drivers of biodiversity: A century of change in the mollusc community of Oneida Lake. *PLoS One* **9**, e101388 (2014).

13. Idrisi, N., Mills, E. L. & Rudstam, L. G. Long-term phytoplankton community dynamics: Oneida Lake (1975–2011). in *Oneida Lake: Long term dynamics of a managed ecosystem and its fisheries* 139–159 (2016).

14. Smayda, T. J. Patterns of variability characterizing marine phytoplankton, with examples from Narragansett Bay. *ICES J. Mar. Sci.* **55**, 562–573 (1998).

15. Sullivan, B. K., Van Keuren, D. & Clancy, M. Timing and size of blooms of the ctenophore Mnemiopsis leidyi in relation to temperature in Narragansett Bay, RI. *Hydrobiologia* **451**, 113–120 (2001).

16. Smayda, T. J. Narragansett Bay Plankton Time Series. Graduate School of Oceanography, URI. https://www.nabats.org/ (2002).

17. Vanni, M. J. & Temte, J. Seasonal patterns of grazing and nutrient limitation of phytoplankton in a eutrophic lake. *Limnol. Oceanogr.* **35**, 697–709 (1990).

18. Hansen, G. J. A. & Carey, C. C. Fish and phytoplankton exhibit contrasting temporal species abundance patterns in a dynamic North Temperate Lake. *PLoS One* **10**, 1–19 (2015).

19. Sharma, S. *et al.* A global database of lake surface temperatures collected by in situ and satellite methods from 1985-2009. *Sci. Data* **2**, 1–19 (2015).

20. Magnuson, J., Carpenter, S. & Stanley, E. North Temperate Lakes LTER: Zooplankton - Madison Lakes Area 1997 - current ver 31. Environmental Data Initiative. https://doi.org/10.6073/pasta/8b265c0300252c87805f26f41e174aa4 (2019).

21. Jacobs, P., Kromkamp, J. C., Van Leeuwen, S. M. & Philippart, C. J. M. Planktonic primary production in the western Dutch Wadden Sea. *Mar. Ecol. Prog. Ser.* **639**, 53–71 (2020).

22. Hillebrand, H. *et al.* Temporal declines in Wadden Sea phytoplankton cell volumes observed within and across species. *Limnol. Oceanogr.* **67**, 468–481 (2022).
