# Supplementary material for: Relationships of temperature and biodiversity with stability of natural aquatic food webs
Source: Nat Commun. 2023 Jun 14;14:3507. doi: 10.1038/s41467-023-38977-6 (PMC10267189; doi:10.1038/s41467-023-38977-6)
Supplement: Supplementary file 3 — Reporting Summary [file 41467_2023_38977_MOESM3_ESM.pdf]

nature portfolio

Corresponding author(s): Qinghua Zhao

Last updated by author(s): May 1, 2023

Reporting Summary

Nature Portfolio wishes to improve the reproducibility of the work that we publish. This form provides structure for consistency and transparency in reporting. For further information on Nature Portfolio policies, see our Editorial Policies and the Editorial Policy Checklist.

Statistics

For all statistical analyses, confirm that the following items are present in the figure legend, table legend, main text, or Methods section.

n/a	Confirmed	
☐	☒	The exact sample size (*n*) for each experimental group/condition, given as a discrete number and unit of measurement
☐	☒	A statement on whether measurements were taken from distinct samples or whether the same sample was measured repeatedly
☐	☒	The statistical test(s) used AND whether they are one- or two-sided<br>*Only common tests should be described solely by name; describe more complex techniques in the Methods section.*
☒	☐	A description of all covariates tested
☐	☒	A description of any assumptions or corrections, such as tests of normality and adjustment for multiple comparisons
☐	☒	A full description of the statistical parameters including central tendency (e.g. means) or other basic estimates (e.g. regression coefficient) AND variation (e.g. standard deviation) or associated estimates of uncertainty (e.g. confidence intervals)
☐	☒	For null hypothesis testing, the test statistic (e.g. *F*, *t*, *r*) with confidence intervals, effect sizes, degrees of freedom and *P* value noted<br>*Give P values as exact values whenever suitable.*
☒	☐	For Bayesian analysis, information on the choice of priors and Markov chain Monte Carlo settings
☒	☐	For hierarchical and complex designs, identification of the appropriate level for tests and full reporting of outcomes
☐	☒	Estimates of effect sizes (e.g. Cohen's *d*, Pearson's *r*), indicating how they were calculated

Our web collection on statistics for biologists contains articles on many of the points above.

Software and code

Policy information about availability of computer code

Data collection	No software was used to collect data.
Data analysis	The CCM and S-map were performed using “rEDM” package (https://cran.r-project.org/src/contrib/Archive/rEDM, version 1.2.3). The multiview distance regularised S-map was performed following Chang et al. (2021). Linear mixed model was performed using lme4 package (https://cran.r-project.org/src/contrib/Archive/lme4,version 1.1.27.1). The missing data points after seasonal average were then linearly interpolated using zoo package (https://cran.r-project.org/src/contrib/Archive/zoo, version 1.8-11). All statistical analyses were performed in R 4.1.2. Programming code for empirical dynamic modelling (EDM) to infer causal links among species, interaction networks, and structural stability of food web is available on GitHub (https://github.com/QZhao16/aquatic.foodweb.stability), are on Zenodo (https://doi.org/10.5281/zenodo.7877806).

For manuscripts utilizing custom algorithms or software that are central to the research but not yet described in published literature, software must be made available to editors and reviewers. We strongly encourage code deposition in a community repository (e.g. GitHub). See the Nature Portfolio guidelines for submitting code & software for further information.

Data

Policy information about availability of data

All manuscripts must include a data availability statement. This statement should provide the following information, where applicable:

- Accession codes, unique identifiers, or web links for publicly available datasets
- A description of any restrictions on data availability
- For clinical datasets or third party data, please ensure that the statement adheres to our policy

All raw data used in this study are publicly available or on request through the paths listed in table S2. Station L4 marine data are archived and available from the British Oceanographic Data Centre BODC (www.bodc.ac.uk) are freely available upon request to Dr. Claire Widdicombe (clst@pml.ac.uk) and Dr. Angus Atkinson (aat@pml.ac.uk) at Plymouth Marine Laboratory.

Research involving human participants, their data, or biological material

Policy information about studies with human participants or human data. See also policy information about sex, gender (identity/presentation), and sexual orientation and race, ethnicity and racism.

Reporting on sex and gender	n/a
Reporting on race, ethnicity, or other socially relevant groupings	n/a
Population characteristics	n/a
Recruitment	n/a
Ethics oversight	n/a

Note that full information on the approval of the study protocol must also be provided in the manuscript.

Field-specific reporting

Please select the one below that is the best fit for your research. If you are not sure, read the appropriate sections before making your selection.

☐ Life sciences ☐ Behavioural & social sciences ☒ Ecological, evolutionary & environmental sciences

For a reference copy of the document with all sections, see nature.com/documents/nr-reporting-summary-flat.pdf

Ecological, evolutionary & environmental sciences study design

All studies must disclose on these points even when the disclosure is negative.

Study description	We first integrated 19 long term planktonic food webs (10-30 years) from two continents. Then, we applied empirical dynamic modelling (EDM) to reconstruct the interaction network and infer species interactions. Next, we computed structural stability and temporal stability in each of the 19 food web. Finally, we examined the relationships between temperature/biodiversity and the two stability indices (structural and temporal stability).
Research sample	For 19 datasets, 18 out of 19 datasets were obtained from the publicly available in open database from North Temperate Lakes, Data Observation Network for Earth, Center for Global Environmental Research, UK Centre for Ecology & Hydrology, Pangaea or Waikato Regional Council. The last dataset from the Western English Channel is achieved by request the British Oceanographic Data Centre. We analyze this comprehensive dataset to quantify the effects of temperature/biodiversity on the two stability indices (structural and temporal stability).
Sampling strategy	This study was a synthesis in aquatic systems. The detail of sampling methodology thus differed among studies. But for each study, organisms were sampled using a similar methodology through time, in which the organisms were generally collected as a vertical tow using mesh nets, and then brought to lab for species identification and measurements. In this study, a total of 19 datasets from 10 to 30 years were compiled, which was sufficient for the synthesis.
Data collection	This study was to synthese 19 datasets of across thousands species, and hundreds of people have been involved in the data collection. The details of investigators and investigating procedures were available in original studies, which were presented in the publications and repositories of following databases (North Temperate Lakes, Data Observation Network for Earth, Center for Global Environmental Research, UK Centre for Ecology & Hydrology, Pangaea, Waikato Regional Council, and British Oceanographic Data Centre).
Timing and spatial scale	All time series data are integrated with seasonally intervals, ranging from 10 to 30 years, over three continents, including lakes, estuary of rivers, and marines.
Data exclusions	We excluded the datasets that were only sparsely sampled (e.g. yearly or semi-annually). We only kept datasets with seasonal

	sampling of all variables (if multiple samplings were conducted per season, e.g. monthly and bimonthly sampling datasets, those were averaged).
Reproducibility	This study was based on analysis 19 datasets over time series, and these datasets came from open-access databases as described above. We did not conduct any experiments. The stratified dataset and the code used to conduct the analyses were both publicly available at Zenodo and GitHub as fully described in Methods section.
Randomization	We used open-access data, and we did not conduct any experiments. The 19 open datasets we used were separately located to different locations. But we treated the sample locations and time (year and season) as random factors in linear mixed model to exclude the potential confounding effect of them. The statistics were fully described in the methods.
Blinding	N/A. Blinding was not relevant to our study because we did not conduct any experiments.

Did the study involve field work? ☐ Yes ☒ No

Reporting for specific materials, systems and methods

We require information from authors about some types of materials, experimental systems and methods used in many studies. Here, indicate whether each material, system or method listed is relevant to your study. If you are not sure if a list item applies to your research, read the appropriate section before selecting a response.

Materials & experimental systems

n/a	Involved in the study
☒	☐ Antibodies
☒	☐ Eukaryotic cell lines
☒	☐ Palaeontology and archaeology
☒	☐ Animals and other organisms
☒	☐ Clinical data
☒	☐ Dual use research of concern
☒	☐ Plants

Methods

n/a	Involved in the study
☒	☐ ChIP-seq
☒	☐ Flow cytometry
☒	☐ MRI-based neuroimaging
